# Walking-related locomotion is facilitated by the perception of distant targets in the extrapersonal space

**DOI:** 10.1038/s41598-019-46384-5

**Published:** 2019-07-08

**Authors:** Sara Di Marco, Annalisa Tosoni, Emanuele Cosimo Altomare, Gabriele Ferretti, Mauro Gianni Perrucci, Giorgia Committeri

**Affiliations:** 1 Department of Neuroscience, Imaging and Clinical Sciences, and Institute for Advanced Biomedical Technologies (ITAB), University G. d’Annunzio, Chieti-Pescara, Italy; 2 Department of Movement, Human and Health Sciences, University of Rome “Foro Italico”, Rome, Italy; 3 Department of Philosophy, University of Florence, Florence, Italy

**Keywords:** Navigation, Human behaviour

## Abstract

The Gibsonian notion of affordance has been massively employed in cognitive sciences to characterize the tight interdependence between hand-related actions, manipulable objects and peripersonal space. A behavioural facilitation effect, indeed, is observed for grasping actions directed to objects located in the ‘reachable’ peripersonal space. Relevantly, this relationship is supported by dedicated neural systems in the brain. The original notion of affordance, however, was directly inspired by real-time interactions between animals and their extended natural environment. Consistently, also the extrapersonal space representation can be significantly modulated by action-related factors, and the brain contains dedicated systems for the representation of topographical space and navigation. Here we examined whether a facilitation effect could be also described for a walking-related action in the far extrapersonal space. To this aim, we employed a go/no-go paradigm requiring subjects to execute a footstep ahead in response to pictures of a virtual reality environment containing objects located at different distances (near, far) and eccentricities (central, peripheral). A walking-related, facilitation effect for distant extrapersonal locations was found, suggesting an automatic trigger of walking by positions that preferentially guide spatial exploration. Based on the parallelism with the literature on micro-affordances, we propose that this effect can be described in terms of “macro-affordances”.

## Introduction

Contemporary cognitive science has been largely influenced by the ethologically-inspired idea that the brain’s functional architecture is organized to reflect the interactive nature of animals’ behaviour in their natural environment^1^. Accordingly, since its inception in 1979^2^, the notion of affordances as perceivable opportunities of action offered by the environment to an animal has been massively employed in experimental psychology and cognitive neuroscience to explain and interpret a series of findings indicating that object- and space-related representations are inextricably linked to action-related representations^3^.

Within this framework, the majority of experimental works have focused on the relationship between hand-related actions, manipulable objects and the reachable peripersonal space. At the behavioural level, for example, a facilitation effect has been observed when a visually-presented manipulable object was associated with the execution of a functionally appropriate hand action (in terms of spatial alignment between the object’s features and the responding hand or of appropriateness of the hand grip)^4,5^. Subsequent studies have shown that this behavioural facilitation is specifically observed when graspable objects are located within the reachable peripersonal space^6,7^, thus indicating that the perception of the affording features of an object (i.e. micro-affordances) is spatially constrained, i.e. it depends on the spatial relationship between the objects and the motor actor.

Therefore, despite the above-mentioned original emphasis on subject-environment interaction, current studies on the issue have specifically focused on reaching/grasping-related affordances or on body-scaled affordances. Classical studies in the field of ecological psychology, for example, have investigated affordances beyond reaching space by employing action judgments and movement adaptations while navigating through apertures/gaps, demonstrating that perception of affordances is body-scaled (e.g. the body size is implicitly used to judge ‘passability’ and to adapt neck and shoulders movement)^8–11^. Within the context of the embodied cognition research tradition, instead, the relevant series of studies by Proffitt and colleagues have employed explicit distance judgments to show that the representation of the large-scale extrapersonal space (i.e. the space that falls far away from our body; see^12^ for a review on different space models and sectors) can be significantly modulated by motor potentialities/resources^13–16^. In particular, it has been shown that contingent factors such as wearing a heavy backpack^13^ or having consumed high caloric substances^15^ as well as stable variables such as the decline in motor functions with aging^16^ can significantly influence the explicit perception of slant and object’s distance within the extrapersonal space. Notably, recent studies have additionally shown that extrapersonal space perception can be significantly modulated by the implicit representation of action possibilities/potentialities of a human-like agent present in the scene, both when it was used as a reference frame for a near/far distance judgment (i.e. other-based judgment^17^) and when it was completely task-irrelevant during self-based judgments^18^. For example, we have shown that the presence of a human-like agent looking at an object brings the observer to judge the object as less distant in the environmental scene.

Finally, it has been recently shown that priming of leg-related actions, such as walking and running, can significantly expand the portion of extrapersonal space judged as near in other-based coordinates^19^ as well as that the peripersonal space is significantly extended during full-body actions such as walking (as compared to standing^20^). Taken together, these results are relevant as they show, for the first time, that visual perception of the extended physical environment is strongly influenced by action possibilities, capabilities and/or intentionality.

However, a still unexplored question is whether there are perceptual factors or variables of the extrapersonal space that directly modulate the planning/execution of a whole-body action such as locomotion. This question appears particularly intriguing because it directly taps into the original Gisbonian notion of affordance as an intrinsic property of the real-time interactions between animals and their natural environment^2^. At the evolutionary level, indeed, all animals appear to have an intimate relationship with their extended natural environment and locomotion appears to be the main form of spatial exploration of the extended environment. In particular, as compared to near extrapersonal locations from which information can be extracted from different sources, locomotion is the only available resource to cover distance and to access information from more distant locations in the extrapersonal space. It is also important to note that, differently from small objects such as faces and tools, environmental scenes/layouts not only develop in depth but also cover a larger portion of the peripheral visual field.

Accordingly, at the neurobiological level, not only it has been shown that the representational structure of scene-related regions of the ventromedial occipito-temporal cortex^21,22^ is primarily defined by the spatial characteristics of an environmental scene such as distance (near, far) and expanse (open, closed)^23^ but also that these regions contain a preferential representation of the peripheral (as compared to the central) visual field^24^. Interestingly, a significant bias towards the periphery of the visual field has also been shown in dorso-medial parietal regions associated with ego-motion^25^ and these two sets of regions (dorso-medial parietal and ventromedial occipito-temporal regions) represent the core components of a medial parieto-temporal network dedicated to the processing of visual information for the purpose of spatial exploration and navigation^26^.

On these bases, locomotion might be preferentially guided by distant and peripheral rather than near and central positions in the extrapersonal space, as peculiar and distinctive features of the environmental layouts.

Following these predictions, here we investigated whether, as for grasping towards centrally-presented objects in the peripersonal space, a facilitation effect could be observed for a walking-related action in response to targets located at further vs. nearer and peripheral vs. central positions of the extrapersonal space. Framed differently, here we investigated whether visual perception of an environmental scene/layout framing objects from a large distance and eccentricity from the observer preferentially affords a walking-related action.

**Figure 1:**
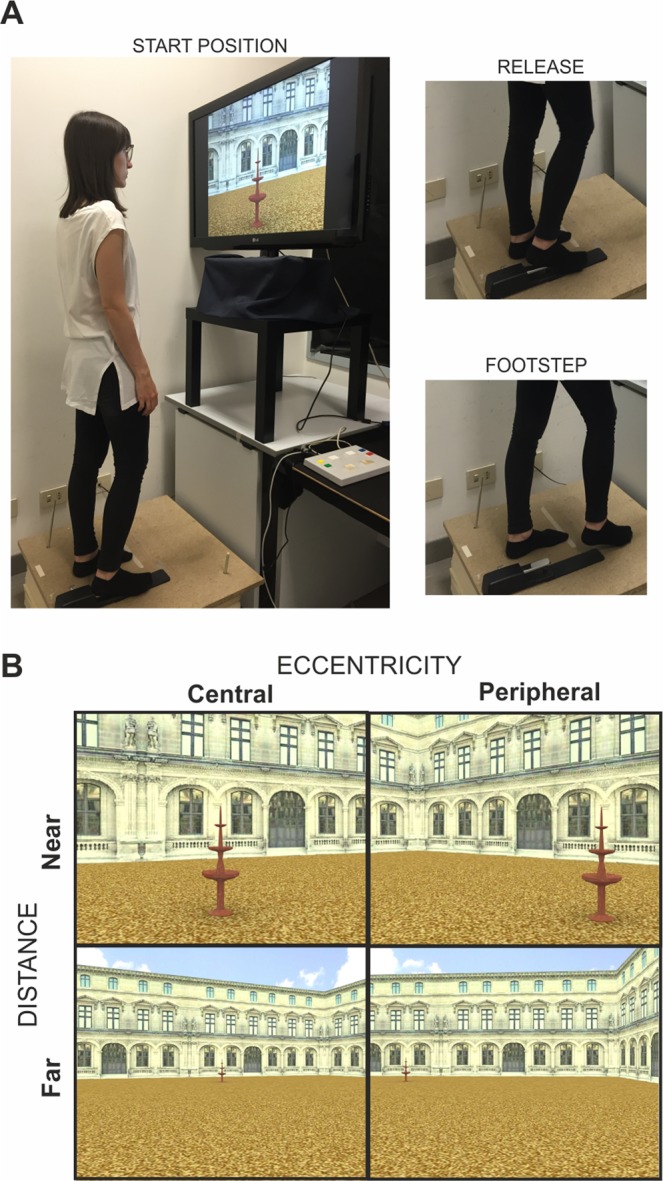
Experimental Design. **(A)** Set up: Experimental subjects were standing on a multi-layer platform positioned in front of a screen covering about 70° of visual angle and holding down a foot-related response pedal with their right heel. The paradigm was a go/no-go task requiring the execution of a footstep ahead or a simple release action in response to pictures of a virtual reality environment containing objects located at different distances (near, far) and eccentricities (central, peripheral) from the observer. The go/no-go task employed in experiment 1 required a visual discrimination of the target object. The go stimulus (fountain, umbrella) was alternated across blocks while the instructed movement (simple release or footstep) was counterbalanced across subjects, with half of the subjects that performed the footstep first and the other half that performed the simple release first. The go/no-go task employed in experiment 2 was based on picture pairs and required an identity judgment (same vs. different) on the whole picture (i.e. go when the target image is identical to the prime image and no-go when different). The two instructed movements (simple release, footstep) were collected in two separate sessions at about 1 month apart. (**B**) The panel shows exemplar stimuli of the two within-subjects visual factors.

To this aim, we presented a series of pictures of a virtual reality environment containing a target object located at different extrapersonal distances (near, far) and eccentricities (central, peripheral) from the observer (see Fig. 1) and required participants to execute a single footstep forward (taken as proxy of walking) or a control action involving a simple pedal release, in response to the target objects.

## Material and Methods

### Participants

The study was conducted on a total sample of 58 right-footed healthy subjects that participated in the study after providing written informed consent in accordance with the ethical standards of the 1964 Declaration of Helsinki.

Experiment 1 was conducted on a total of 30 participants (mean age: 22; 13 males) and the execution order of the instructed movements (simple release, footstep) was manipulated between groups: half of the subjects performed the footstep action first (Group 1) and the other half performed the release action first (Group 2).

Experiment 2 was conducted on a group of 18 participants (mean age: 25.2; 9 males), with the two movements collected in separate sessions.

The remaining 10 participants (mean age: 27, 5 males) were enrolled for a control study on explicit perception of the object’s distances employed in the two main studies of the work.

All participants had normal or corrected-to-normal vision and were naïve as to the purposes of the experiments. The protocol was approved by the Ethics Committee of G. d’Annunzio University of Chieti, Italy.

### Experiment 1: stimuli, apparatus and procedure

Stimuli included a selection of pictures from a virtual reality environment, created by means of a 3D modelling software (3D Studio Max 4.2, Autodesk, Discreet) representing a square arena of a three-winged palace with a beach umbrella or a marble fountain of similar size and color positioned in front of the central wing.

Before the beginning of the experiment, a movie simulating a regular-speed walk across the environment was shown to subjects in order to get them familiar with the environment.

Subjects were instructed to stand on a multi-layer platform positioned in front of a 42″ screen covering about 70° of visual angle (57 cm distance) and to hold down an in-house customized piano pedal with their right heel. A series of pictures of the environment were then presented in which both the distance (near, far) and the eccentricity (central, peripheral) or the target object (the umbrella or the fountain) was manipulated across trials and subjects were instructed either to simply release the foot pedal with the heel of their right foot (while keeping the foot’s front on the ground) or to release the pedal and execute a footstep ahead at the onset of the picture and to get back in the starting position (see Fig. 1 for the experimental set-up).

The subjects’ task was to respond at the onset of the picture when it contained the fountain or the umbrella (go/no-go task) with the “go” stimulus alternated across blocks. The reaction time associated with the foot pedal release was recorded on each trial. Importantly, the go/no-go task was exclusively employed to ensure that participants’ attention was focused on the target object without an explicit coding of its spatial properties. However, since the task required a perceptual discrimination of the target stimuli (umbrella vs. fountain), we expected that the lower perceptual salience of the targets in far and peripheral positions (compared to near and central) would induce a perceptual difficulty confound (i.e. more difficult perceptual discrimination for far and peripheral vs. near and central targets). Therefore, the simple release action was included in the design as to control for both the perceptual difficulty, as well as for the basic aspect of the footstep action (including the proprioceptive inputs from the pressure/release of the foot pedal). We made use of the simple pedal release action as a baseline measure that included the perceptual factors of identifying central vs. peripheral targets. This baseline measurement was subtracted from the reaction time associated with taking the full forward footstep motion in each condition, resulting in a value we dubbed the “walking cost.” In other words, the walking cost is the difference between the mean releases times associated with the footstep action vs. the simple release action for each of the four conditions of distance and eccentricity. If a lower walking cost is observed in response to targets positioned in further vs. nearer and peripheral vs. central locations of the extrapersonal space, then we will have identified a walking facilitation effect for far and peripheral targets, akin to the facilitation of grasping actions for targets presented centrally in peripersonal space.

Concerning the instructed movements, it is worth noticing that during the simple release action (i.e. control action) the leg remained still on the response pedal and the body weight loaded on the (left) non-responding foot, while during the footstep action, the participants were instructed not only to move the leg for a step length but also the entire body ahead as if they were about to start walking. As a result, during the footstep action, the body weight was shifted from the non-responding left to the responding right foot and this could be correctly executed without falling off the platform or running into the equipment. Notably, we are aware that the footstep action is not as natural as walking but, as noted in the Introduction, in the current work we consider this action as a valid proxy for walking because there are basic postural, muscular and kinematic elements that are shared between the two actions.

Target distance and eccentricity were manipulated across trials by framing the object at 8 possible distances (from 5 to 8 virtual meters for the near condition and from 28 to 40 virtual meters for the far condition) and at 2 levels/amplitudes of visual angle with respect to the observer’s central point of view (0° for the central condition and from 15° to 30° for the peripheral condition). Specifically, the object occupied a fixed position in the environment and the pictures framing the target object were rendered by moving the camera at different extrapersonal distances along a vector connecting the camera with the target object and by rotating the camera at different degrees of visual angle. This ensured that, for each selected distance, the peripheral and central objects were equally distant from the observer. The side of presentation of the target object for the peripheral condition (left, right) was balanced and randomized across the different extrapersonal distances. Distances were expressed in virtual meters (which have been estimated as approximately doubled with respect to real distances in the current environment^18^) and were selected with particular reference to the Grüsser’s distinction between a near-distant and a far-distant extrapersonal action space^27^. In particular, near and far locations were selected to fall very far from the proposed ~8 meters (i.e. 16 virtual) boundary defining a near- vs. far-distant extrapersonal action space^18,27^.

Each participant completed 8 randomized blocks for each instructed movement (i.e. 8 blocks for the footstep action and 8 blocks for the simple release action), with each block including 32 go pictures and 8 no-go pictures. Participants did not receive any feedback about their accuracy performance but blocks with more than two recorded errors were repeated at the end of the session. In order to make the experiment as more ecological as possible, moreover, no specific instructions about fixation were given to the participants.

Pictures were alternated each 3000–4500 ms with a white fixation cross on a black background and were presented until a response was recorded for a maximum of 2500 ms.

Before starting the experiment, each participant was extensively trained on the task until the movement was correctly executed without the need of looking down at the feet and the starting position retrieved before the onset of each picture.

Stimulus presentation and response collection were controlled by a customized software based on Cogent 2000 and implemented in Matlab (The MathWorks Inc., Natick, MA, USA).

### Experiment 2: stimuli, apparatus and procedure

Experiment 2 was based on the same set of visual stimuli (i.e. pictures from a virtual reality environment containing a target object located at different extrapersonal distances and eccentricities from the observer) and behavioural responses (i.e. simple release, footstep) employed in experiment 1 but the two instructed movements were collected in two separate sessions (at about 1 month apart) and using a different go/no-go paradigm. The paradigm employed in experiment 2, in particular, involved the presentation of pictures pairs (i.e. prime and target image) and required subjects to execute the instructed movement only when the target picture was identical to the prime picture and to refrain the response when the pictures were different (note that the two images for the no-go trials belonged to different distance’ categories, i.e., near/far and vice versa, thus resulting in a very easy discrimination). Therefore, the current paradigm was aimed at avoiding the potential perceptual difficulty confound associated with the discrimination of objects at different distances and eccentricity (i.e. more difficult perceptual discrimination for far and peripheral vs. near and central objects). Each picture was presented for 700 ms with an SOA of 500 ms between the prime and target picture and an ITI of 2000 ms between each pictures pair. As indicated above, the response was provided at the onset of the target picture and the reaction time associated with the foot pedal release was recorded on each trial.

Each participant completed 2 experimental sessions (simple release and footstep) collected at about 1 month apart and each session included 6 blocks with 32 go trials (target picture equal to prime picture) and 8 no-go trials (target picture different from prime picture).

Data for the two instructed movements were collected in separate sessions to avoid potential order effect between execution of the two actions (e.g. simple release action executed as a sort of inhibited/blocked footstep when collected immediately following the footstep session). All participants were instructed to maintain fixation on the central fixation cross throughout the experiment.

As for experiment 1, before the beginning of the experiment, a brief training was performed on each participant in order to ensure that the two instructed movements were correctly executed.

The same experimental set-up of experiment 1 was used (i.e. pictures projected on a 42″ screen covering about 70° of visual angle) but stimulus presentation was controlled by the E-Prime software and the foot-related responses were recorded through a foot-pedal response system connected with the E-Prime software.

## Results

### Experiment 1

Analysis of the accuracy performance in experiment 1 indicated that the number of repeated blocks (i.e. blocks with more than two recorded errors) was very low (9 repeated blocks over a total of 480 blocks: 1.8%) with an average number of errors per block of 3.1 (standard deviation: 0.4).

**Figure 2:**
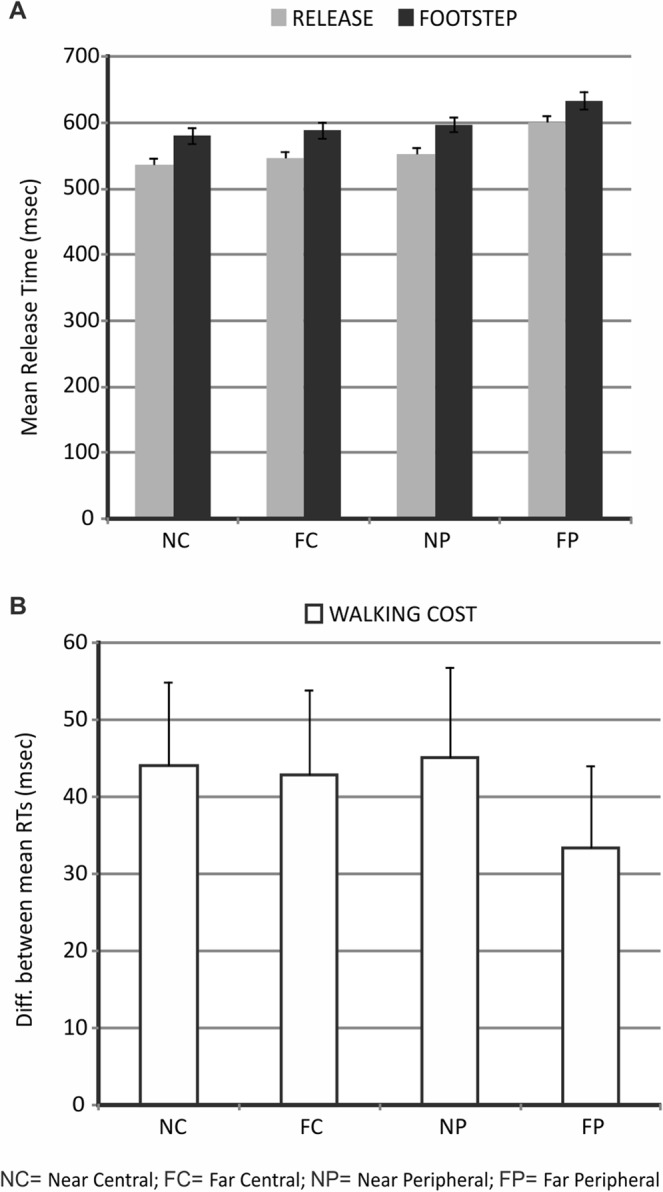
Results of Experiment 1. (**A**) The graph displays the release times associated with the execution of the footstep action and the simple release action as a function of target distance (near, far) and target eccentricity (central, peripheral) mediated across the two groups that performed the two instructed movements with an inverted order. (**B**) The graph displays the “walking cost” (i.e. the difference between the mean release time associated with the footstep action vs. the mean release time associated with the simple release action) as a function of target distance and target eccentricity.

The “error” blocks were then removed from the dataset and analysis of the release times was conducted on the original blocks with less than two recorded errors plus the blocks which were repeated at the end of the session. As illustrated in Fig. 2 (panel A), the results showed both a general increase of the release times for the execution of the footstep action vs. the simple release and a progressive increase of the release times from the near-central condition to the far-peripheral condition. Notably, a similar trend was observed in the two groups with an inverted order of the two instructed movements. This impression was statistically confirmed by the results of a mixed ANOVA with movement order (group that performed the footstep action first, group that performed the simple release first) as a between-subjects factor and movement type (simple release, footstep), target distance (near, far) and target eccentricity (central, peripheral) as within-subjects factors. The ANOVA results, indeed, indicated a no significant effect of movement order (main effect of movement order and interactions between movement order and movement type, target distance and target eccentricity, all p > 0.05) but a significant main effect of movement type (F_(1,28)_ = 15.42, p < 0.001), target distance (F_(1,28)_ = 233.7, p < 0.001) and target eccentricity (F_(1,28)_ = 386.2, p < 0.001), as well as significant interactions between movement type and distance (F_(1,28)_ = 7.02, p = 0.01; Cohen’s d = 0.5; LSD post-hoc comparisons: footstep far > footstep near > release far > release near, all p < 0.05) and between distance and eccentricity (F_(1,28)_ = 167.1, p < 0.001; LSD post-hoc comparisons: FP > NP > FC > NC, all p < 0.05).

Therefore, in addition to a general cost for the execution of the more complex footstep action, longer release times were observed for targets in further and more peripheral positions and this pattern was independent of whether the footstep was executed before or after the simple release.

As noted above, however, since these results were attributed to a perceptual confound associated with perceptual discrimination of the target object, we next examined modulations by extrapersonal distance and eccentricity with respect to a measure defined as “walking cost”, corresponding to the difference between the mean release times associated with the footstep action vs. the simple release action for each of the four conditions.

Data inspection indicated that far and peripheral locations were associated with a relatively smaller “walking cost” with respect to near and central locations. To examine whether this impression was statistically confirmed, a mixed ANOVA was conducted on the “walking cost” with movement order as a between-subjects factor and target distance (near, far) and target eccentricity (central, peripheral) as within-subjects factors. Besides replicating the non-significant effect of movement order already observed in the former ANOVA, this analysis indicated a significant main effect of target distance (F_(1,28)_ = 7.03, p = 0.01; Cohen’s d = 0.5) and a marginally significant distance by eccentricity interaction (F_(1,28)_ = 3.4, p = 0.07; LSD post-hoc comparisons: FP < NP, FC and NC, all p < 0.05).

Therefore, consistent with our predictions, target objects positioned in further and, to some extent, more peripheral locations were particularly spared from the extra cost of moving the foot forward.

### Experiment 2

As for experiment 1, data from experiment 2 were analyzed as a function of target distance and target eccentricity. However, since the go/no-go task (go when the target image is identical to prime image and no-go when different) was specifically aimed at avoiding the perceptual difficulty confound associated with the target object discrimination observed in experiment 1, the effect of target distance and target eccentricity was directly estimated on the release times associated with the two instructed movements.

**Figure 3:**
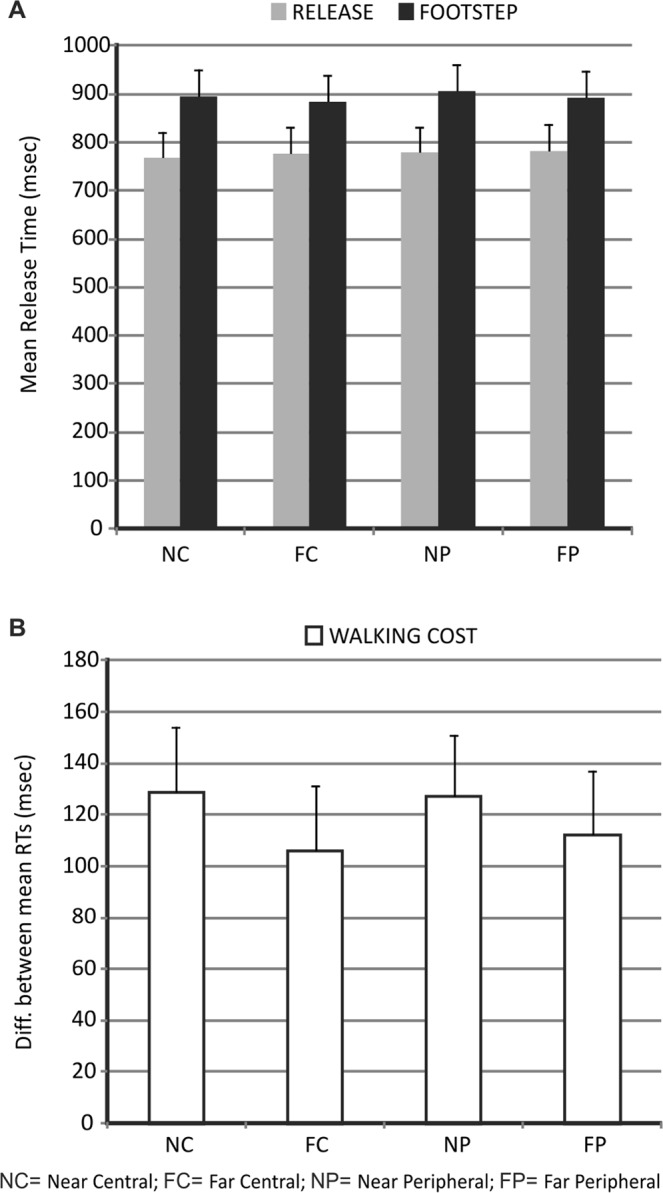
Results of Experiment 2. (**A**) The graph displays the release times associated with the execution of the footstep action and the simple release action as a function of target distance (near, far) and target eccentricity (central, peripheral). (**B**) Data are displayed as in Fig. 2B.

As illustrated in Fig. 3A, in addition to a general increase of the release times for the execution of the footstep action vs. simple release, the results of experiment 2 showed relatively shorter release times for execution of the footstep action in response to objects positioned in further vs. nearer locations. Statistically, this was confirmed by a significant main effect of movement type (F_(1,17)_ = 23.9, p < 0.001) and a significant movement type by distance interaction (F_(1, 17)_ = 7.72, p = 0.01; Cohen’s d = 0.67) in a repeated-measures ANOVA with movement type (simple release, footstep), target distance (near, far) and target eccentricity (central, peripheral) as factors. Post-hoc comparisons (LSD) on the interaction confirmed a significant advantage for execution of the footstep action, and not the simple release action, in response to target objects in far vs. near locations (far vs. near, footstep action: p = 0.01; far vs. near, simple release: p = 0.22).

Therefore, consistent with our predictions, data from experiment 2 showed a facilitation effect, in the form of a classical reaction-time advantage, for a walking-related as compared to a control action in response to targets positioned in further vs. nearer locations of the extrapersonal space (also see Fig. 3B for a plot of the “walking cost” in the different conditions).

As far as the accuracy performance, instead, data were analyzed by conducting an ANOVA on the percentage of correct responses during the “go” trials with movement type (simple release, footstep), target distance (near, far) and target eccentricity (central, peripheral) as factors. Consistent with our expectations, the accuracy performance was quite high during the experiment (mean accuracy = 99%) with no significant difference between conditions (all p > 0.05). Moreover, the false alarms rate, defined as the percentage of trials with a recorded response during the no-go trials, was very low (2.6%).

As a final control analysis, in a separate group of subjects we examined whether the employed distances were perceived correctly by requiring an explicit categorical judgment of the distance (near vs. far). The results showed that the perceived category corresponded to our categorization of the distances (mean accuracy = 97%, 3% of mismatch trials predominantly occurring during judgments of the most distant position within the near category).

## Discussion

Summing up, based on previous behavioural and neuro-functional evidence, here we investigated whether far and peripheral spatial locations within the environmental extrapersonal space are able to facilitate a walking-related action.

The results showed a facilitation effect, both in the form of a reduced cost for moving the foot forward vs. simply releasing the foot pedal (experiment 1) and as a more classical reaction-time advantage for a walking-related action as compared to a control action (experiment 2), in response to target positioned in distant locations of the extrapersonal space.

Importantly, since the employed tasks did not require an explicit coding of the object’s spatial properties, the walking-related facilitation effect observed in this study was completely implicit.

Notably, moreover, since in the current study we were specifically interested in the study of walking-related modulation in the extrapersonal space, all targets objects were presented to fall beyond the reaching space. Therefore, although all the positions would require locomotion to be reached, the crucial result is that further positions were more relevant than nearer positions for guiding locomotion.

As far as the eccentricity factor, however, the analysis of the release times for both the movement types in experiment 1 have highlighted a significant main effect of eccentricity and a significant eccentricity by distance interaction trivially due to a perceptual difficulty confound (i.e. higher release times for far and peripheral spatial locations), while interestingly the relevant data on the walking cost showed only a marginally significant eccentricity by distance interaction towards the opposite direction (i.e. lower release times for far and peripheral spatial locations). Data of experiment 2, moreover, have showed no significant effect of target eccentricity. Therefore, albeit the neurophysiological literature reported in the introduction suggested a role for the eccentricity factor in our study, we failed to observe a robust effect of this factor. This might be explained by a series of methodological factors including the specific features of the experimental task which was not particularly challenging with respect to the coding of the peripheral information (e.g. no obstacles were included in the environment), the lack of fixation monitoring or the limited extension of the peripheral visual stimulation. Further studies using an immersive virtual reality environment with a wider stimulation of the visual periphery and an accurate control of fixation might hopefully clarify this issue.

In our view, the present finding, which closely follows the original^2^ and more recent^1^ definition of affordance as a general property of the real-time interactions between animals and their extended natural environment, is notable for two main reasons. First, because, differently from grasping actions, whole-body actions, such as walking, have never been studied in relation to spatial coding and in particular to extrapersonal space coding, while it is incontrovertible that these actions typically operate within this space portion. Second, because, for the first time, they investigate the nature of the mechanisms underlying spatial coding of the extrapersonal space from a motor perspective, and in particular from the perspective of the afforded action, thus paralleling the extensive research tradition on grasping-related affordance. In particular, it has been extensively demonstrated that features of an object (the so-called micro-affordances) are able to potentiate specific movements (e.g.^5^) that are best suited for interacting with the object itself (reach-to-grasp motor acts) and that such an effect is modulated by the object location, i.e. it is observed only when the object is located within the reachable peripersonal space^6,7^. Here we found an analog of the motor facilitation effect found in these studies, but within the extrapersonal space: while the specific features of an object located in the peripersonal space automatically triggers a reach-to-grasp action, the specific spatial locations of objects within the extrapersonal/environmental space facilitate a walking action. Therefore, based on the parallelism with the behavioural literature on micro-affordances, we suggest the term of “macro-affordances” to describe such a relationship.

From a theoretical point of view, the affordance in question can be surely interpreted at an intuitive level by hypothesizing that when participants anticipate walking further their action starts faster or, in other words, that the more a condition (in this case the far condition) requires an action (i.e. walking more), the more that action will be facilitated. However, along with this basic interpretation of the effect, which is based on the “reachability”, of the target object (i.e. objects in further positions require more or faster walking to be reached), we propose that far locations also automatically activate mechanisms for spatial exploration, which in turn facilitate the walking action. In particular, since distant locations of the extrapersonal space mark the boundaries of the visually defined surrounding environment, and thus constitute the peculiar perceptual features that define the spatial layout as a stimulus, one appealing hypothesis for the behavioural facilitation observed in this study is that the execution of a walking-related action is intrinsically linked to the representation of the spatial layout. That is to say, while a reach-to-grasp action, triggered by the features of an object, is best suited for interacting with the object itself, a walking action, afforded by far and, to some extent, peripheral locations of a spatial layout, is best suited for exploring the environment in all its extent through locomotion. Therefore, we speculate that the facilitation observed might be mostly guided by the nature of the environmental stimulus itself and that such effect might have an evolutionary significance/basis associated with the exploration of the surrounding environment.

With particular reference to the spatial variables examined in our study, we manipulated the radial dimension by selecting metric distances on the basis of a previous study conducted in our lab using the same virtual reality environment and showing a threshold of ~16 virtual meters (corresponding to ~8 real meters) between the near and far extrapersonal space^18^. Notably, such a near/far extrapersonal threshold also corresponds to the sub-division between a near-distant and a far-distant action extrapersonal space proposed by Grüsser^27^ on the basis of a blind-walking paradigm in which subjects were trained to walk over an environmental path. Interestingly, our results confirm such a distinction between a near and a far extrapersonal space since they differently trigger a walking action. More importantly, they offer the brand-new motor evidence that the far extrapersonal space is intrinsically linked to action and specifically to a whole-body action like walking, thus complementing the action-related effects observed during the perception of the extended physical environment^28^. Our observation also helps to better define the properties of the extrapersonal space. Inspection of the traditional neuropsychological literature on the perception of 3-D visual space reveals, indeed, that this space has been typically considered in relation to oculomotor exploration^29^ while being never explicitly or directly associated with the walking action. The Grüsser’s far-distant space is essentially a visual space, with a partial contribution of vestibular and auditory signals^27^. In a similar vein, according to the 3-D space partitioning model proposed by Previc^12^, the space portion extending from approximately 2 to 30 meters from the body, which is referred to as “action extrapersonal”, is mainly concerned with head, eyes and trunk movements, while lower limbs have been associated with the sector of space extending to the outermost boundaries of the visual field, which is labeled as “ambient extrapersonal”. Concerning the functional specialization of these different space sectors, however, while the action extrapersonal space has been explicitly associated with spatial orienting and navigation, the ambient extrapersonal space has been only associated with maintenance of a correct upright stance with respect to gravitationally references during locomotion. Therefore, classical models have somehow detached lower limbs movements from their role in active exploratory behaviour within the extrapersonal space. Interestingly, however, an emerging idea in this very same literature is that spatial interactions in 3-D space are also differently regulated at the level of the brain neurochemical systems, with a specific role of the noradrenergic system in peripersonal functions, including sensory-motor coordination during consummatory behaviours, and of the dopaminergic system in exploratory behaviour in the extrapersonal space^30^. We speculate that this notion of a dopaminergic system that “facilitates behaviours in extrapersonal space, toward which active, psychomotor drive mechanisms are directed”^12^ is reminiscent of a system that is associated with walking/locomotion and that might be triggered by distant stimuli. Accordingly, both animal and clinical studies consider the locomotor exploratory profile as an important behavioural index, with particular reference to the dopaminergic system^31^.

Future studies will be needed, however, to address several questions that remain open from this study. With respect to the question concerning the nature of the environmental stimulus, in particular, it would be relevant to investigate whether the facilitation effect is preferentially associated with stable objects in the environment (i.e. landmarks) or whether it is independent of the object’s navigational features, and whether it can be modulated by incentive-motivational variables associated with the rewarding nature of the environmental stimuli that might increase the psychomotor drive toward them. It would also be relevant to determine whether the facilitation effect observed in our study is associated with the visual perception of the spatial positions and execution of the walking action with respect to specific reference frames. Since in the current study participants were required to perform a forward footstep thus always maintaining a frontal heading aligned to the starting position, the position of objects in the environment remained unvaried (i.e. central or peripheral) with respect to the trunk orientation. Therefore, it remains to be tested whether, as for the peripersonal space^32^, the trunk is the most relevant body part to which the extrapersonal space representation is anchored. Finally, the modulation of distance on the walking-related action was observed despite the spatial scale of the virtual reality environment was more consistent with the definition of a “vista space”, i.e., a space that can be visually apprehended from a single location or with little exploratory movements (viewed within a glimpse), rather than with a proper “navigational space”, i.e. a space that can be experienced only through locomotion^33^. Therefore, a question worth exploring is whether the facilitation effect observed in this study could be even more evident when requiring a walking-related action towards imagined locations within a complex topographical environment.

To conclude, our findings provide an important extension of the general notion of affordance, which goes beyond the notion of micro-affordance, by offering the one of ‘macro-affordance’.

Such a notion denotes the evidence that not only the features of an object located in the “reachable” peripersonal space, but also the spatial position of objects in the far extrapersonal space is able to provide the perceiver with the perception of a possible action. In other words, our results suggest that far extrapersonal space is “ready to walk”.
